# Assessing Child Obesity and Physical Activity in a Hard-to-Reach Population in California’s Central Valley, 2012–2013

**DOI:** 10.5888/pcd12.140577

**Published:** 2015-07-23

**Authors:** Sara E. Schaefer, Rosa Camacho-Gomez, Banefsheh Sadeghi, Lucia Kaiser, J. Bruce German, Adela de la Torre

**Affiliations:** Author Affiliations: Rosa Camacho-Gomez, Banefsheh Sadeghi, Adela de la Torre, Center for Transnational Health, University of California, Davis, California; Lucia Kaiser, Department of Nutrition, University of California, Davis, California. J. Bruce German, Foods for Health Institute, University of California, Davis, California.

## Abstract

**Introduction:**

In California’s agricultural Central Valley, the rate of childhood obesity is higher than the national average. Adequate physical activity contributes to obesity prevention and its assessment is useful to evaluate the impact of interventions.

**Methods:**

Niños Sanos, Familia Sana (Healthy Children, Healthy Family [NSFS]) uses community-based participatory research to implement an intervention program to reduce childhood obesity among people of Mexican origin in the Central Valley. Anthropometric measurements were conducted on more than 650 children enrolled in NSFS. Physical activity data from a subgroup of children aged 4 to 7 years (n = 134) were collected via a wearable accelerometer.

**Results:**

Children were classified on the basis of age and sex-adjusted body mass index as healthy weight (57.7%); overweight (19.3%), or obese (23%). Logistic regression showed that moderate to vigorous physical activity (MVPA) was associated with a child’s likelihood of having a healthy BMI (odds ratio: 1.03; 95% CI, 1.01–1.05; *P* = .017).

**Conclusion:**

NSFS’s community-based participatory approach resulted in successful use of a commercial electronic device to measure physical activity quantity and quality in this hard-to-reach population. Promotion of adequate daily MVPA is an appropriate and necessary component of NSFS’s childhood obesity prevention strategy.

## Introduction

Latinos are the largest and fastest growing ethnic group in the United States and have among the highest rates of overweight and obesity (1,2). Compared with other ethnic groups, Latino children and adolescents in the United States are disproportionately affected by obesity (3,4), which suggests the need to understand causes of obesity among US Latinos so that culturally appropriate interventions can be effectively designed and delivered (5).

Increasing childhood obesity highlights the importance of family-centered interventions (6–8). For example, Barkin et al showed that a culturally tailored intervention involving Latino parents and children improved short-term early growth patterns of the children (6). Niños Sanos, Familia Sana (Healthy Children, Healthy Family [NSFS]) is a 3-year multifaceted intervention targeting California’s populations of Mexican origin where the rate of childhood obesity exceeds the national average (9). The main research goal of NSFS is to evaluate the effectiveness of this community-based intervention on reducing obesity in children of Mexican origin (9). This educational and behavioral change intervention includes 30 family nights (nutrition and physical activity lessons) for parents, classroom nutrition education for children, and an enhanced physical activity program for children. A key component of the intervention is a monthly $25 fruit and vegetable voucher to each family, designed to address the reality that parents make food choices for young children but may not have the resources to make healthy choices. This article describes a substudy in the NSFS intervention that involved assessing the physical activity levels of participating children with accelerometers.

Establishing regular physical activity during childhood helps prevent obesity and related diseases (10). National guidelines recommend that children engage in moderate to vigorous physical activity (MVPA) for a minimum of 60 minutes every day (11,12). To evaluate the effectiveness of interventions and policies to increase physical activity among children, reliable measures of MVPA are essential. Accelerometry objectively measures the frequency, intensity, and duration of physical activity (13,14). These wearable devices are a reference method for measuring children’s activity in their normal daily lives (15–17). Until recently, acquiring accurate and practical instruments to measure physical activity was a challenge for large-scale research efforts, especially for research targeting children (16,18,19). Self-report methods are not feasible for children and direct observation is not feasible for measuring large groups (17). Furthermore, the high cost of electronic accelerometers is often a barrier to studies of large groups. Our objective was to assess the effects that using commercially available accelerometers would have on measuring the physical activity patterns of children of Mexican origin and on their body mass index (BMI).

## Methods

Approval for this research was granted by the institutional review board at the University of California, Davis (UCD). The NSFS program uses a community-based participatory research (CBPR) approach (20) in 2 Central Valley towns, Firebaugh and San Joaquin, in Fresno County, California. These towns are demographically and geographically similar; both are rural, have a population that is more than 90% of Mexican origin, and have a primary employment base that is agricultural. Community members in both towns are largely monolingual Spanish-speaking immigrants with low income and very low educational levels who rely on seasonal employment. All age-eligible children (aged 3–8) were recruited for this study, independent of ethnic/racial status, because in both towns, more than 90% of the children are of Mexican origin (9). More than 650 children were enrolled. The NSFS methodology is described in detail elsewhere (9). This article presents physical activity data collected on a subsample of children (n = 134) aged 4 to 7.

From April 2012 through January 2013, anthropometric measurements, including weight, height, abdominal circumference, and skinfold thickness (triceps and subscapular), were collected on eligible children. Trained local staff and UCD students collected the measurements following procedures described in the Anthropometric Standardization Reference Manual (21). A digital scale by Seca (model 874) was used to weigh participants to the nearest 0.1 kg. A stadiometer (Seca 213) was used to measure height in centimeters (cm) to the nearest 0.1 cm. Training and standardization manuals were developed and used to ensure precision and standardization of data. All the surveyors involved in measurement are standardized to ensure accuracy and validity of measurements.

The technical error of measurement is used to calculate inter-observer and intra-observer errors. For the inter-observer error, the surveyors compare their results with the lead anthropometrist’s results. Standardization occurs before each anthropometric data collection period. Child BMIs, age- and sex-specific percentiles, and *z*-scores were calculated by using the Centers for Disease Control and Prevention references (22). Children were classified as healthy (BMI <85th percentile), overweight (BMI ≥85th percentile and <95% percentile) or obese (BMI ≥95th percentile).

**Figure 1:**
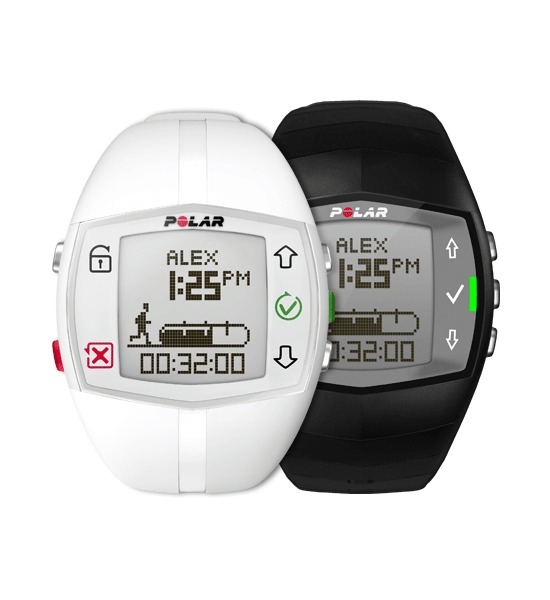
Polar Active accelerometer watches used for 24/7 activity measurement for 134 children enrolled in Niños Sanos, Familia Sana (Healthy Children, Healthy Family), Central Valley, California, 2012–2013.

Trained staff interviewed parents to collect demographic and economic data, including several acculturation variables (language and time in the United States) (23). Physical activity was measured by using the Polar Active, a uniaxial accelerometer that records vertical acceleration and indicates the intensity of physical activity associated with locomotion (Figure 1). This wrist-worn, waterproof device provides continuous 24-hour measurement of time in 5 intensity zones (very easy, easy, moderate, vigorous, and very vigorous), sleep duration, number of active steps, and total energy expenditure. Data are recorded in 30-second periods over the duration of use. The development and validation of this device were previously described (24,25). Briefly, physical activity measurement by the Polar Active shows high correlation with the measurement of oxygen consumption by children (*R* = 0.93) (25). A protocol developed to collect physical activity data on a subsample of children enrolled in the NSFS program excluded those younger than 4 years of age because of differences in the nature of movement by younger children (26). A qualitative pilot of the device protocol was carried out among children of the same age in a local rural community both culturally and socioeconomically similar to the NSFS study population. The results revealed high acceptability and compliance via early saturation.

The Polar Active has user feedback features on its face. These features include an activity bar that represents the user’s time in MVPA and an animated figure indicating the user’s current activity intensity. Such features may engage users in their activity levels and skew normal behavior; in research studies participants should be blinded to the measured variable to prevent bias. To address this issue, researchers followed a protocol of instructing children to go about their normal activities and did not explain the meaning of the accelerometer watch features. Accelerometer watch settings were locked before distribution so that children could not access data stored in the device. During the months of April through August 2012, children enrolled in NSFS aged 4–7 years were asked to use the watch-like device for 9 consecutive days, allowing for the collection of up to 7 complete days of data. Our CBPR approach (9) used a combination of collaborative strategies between research and field staff, school staff, and health promoters to distribute devices to individual children during community events, health fairs, home visits, and field office appointments. At distribution, children and their parents were verbally instructed not to remove the waterproof device during the period of use, including while sleeping and during water-based activities such as bathing and swimming. Parents were provided a diary to note any removals, including the time, duration of, and reason for removal. On the last day of assigned use, parents were instructed to return the device to the field office in Firebaugh. Alternatively, program staff retrieved the device from participants’ homes. Data analyses were conducted in SPSS version 22 (IBM, Inc). Descriptive statistics were calculated for all variables (mean, standard deviation, range). Logistic regression models of healthy versus overweight or obese status were used to explore sex and age interactions in relation to activity variables (daily MVPA, steps, energy expenditure, and sleep duration) to examine the potential impact of activity levels on obesity patterns.

## Results

A total of 185 children used accelerometers and 175 returned devices with data. After excluding cases of low compliance, defined as the collection of fewer than 3 days of valid physical activity data, data for 134 children were included in final analyses (64 boys and 70 girls). For these children, average household size was 5 with a standard deviation (SD) of 1.36 (range, 2–11) people. Assessment of acculturation at the household level revealed a score of −2.19, SD, 1.64 (range, −4 to +3). Based on the original acculturation scale by Cuellar and colleagues (23), 81% of households in this sample are in the least acculturated group. Socioeconomic and demographic characteristics of parents (n = 245) included an average age of 36.6 years, average level of education of 8.7 years, and average time in the United States of 18 years. Spanish is the primary language for 78.9%, and 54.2% had health insurance. A valid day was defined as at least 20 hours of collected data (to avoid days when the device was removed for excessive periods of time). There was a 72% compliance rate with the protocol among children in our study. We assessed reactivity (change in behavior attributed to device use) by conducting *t* tests of MVPA time on the first and last valid measurement days; no differences were found.

Anthropometric characteristics of this sample population (Table 1) show no significant differences between boys and girls, so the combined data are presented. Age-adjusted BMI calculations classified 19.3% of children as overweight and 23% as obese. Physical activity data (Table 2) show no differences in physical activity variables (MVPA, sedentary activity, steps, energy expenditure) measured during the school year and summer. We used *t* tests to examine differences between boys and girls. Compared with girls, boys had greater daily MVPA, daily energy expenditure and steps per day, and slept fewer hours per night (Table 2). Correlation analysis indicated that among boys only, sleep duration was inversely associated with BMI (*R* = −0.25); no associations were seen among girls or among both sexes combined.

**Table 1:** Anthropometric Characteristics of 134 Child Participants in Niños Sanos, Familia Sana (Healthy Children, Healthy Family), Central Valley, California, 2012–2013

Characteristic	N^a^	Mean (SD)	Range
Age, y	134	5.62 (1.03)	2.80 to 7.90
Weight, kg	133	21.98 (4.85)	13.55 to 40.93
Height, cm	133	113.17 (8.41)	97.45 to 156.20
BMI	133	17.12 (2.27)	12.90 to 25.10
BMI, percentile	133	71.92 (26.04)	0.20 to 99.90
BMI *z*-score	132	0.79 (1.03)	−2.33 to 3.06
Abdominal circumference, mm	119	56.09 (7.87)	23.18 to 80.85
BF % triceps	120	12.14 (4.64)	5.50 to 31.50
BF % subscapular	119	8.62 (4.77)	3.25 to 30.50

Abbreviations: BF, body fat; BMI, body mass index.

a Sample size (N) varies because some children refused to be measured.

**Table 2:** Physical Activity of 134 Child Participants in Niños Sanos, Familia Sana (Healthy Children, Healthy Family), Central Valley, California, 2012–2013

Variable	Mean (SD)		*P* Values^a^	Range	
	Boys	Girls		Boys	Girls
Days collected, n	6.6 (0.1)	6.6 (1.0)	NA	3.0–7.0	3.0–7.0
MVPA, min/d	86.2 (29.2)	68.7 (25.7)	<.001	25.4–163.1	22.2–140.5
SEDA, min/d	867.8 (61.1)	860.5 (57.8)	.812	650.8–1,023.7	614.0–990.8
Sleep duration, min/d	485.5 (56.2)	506.9 (56.9)	.034	263.9–723. 0	368.8–788.5
Energy expenditure, kcal/d	1,546.7 (248.3)	1,369.5 (187.0)	<.001	1,178.0–2,453.1	1,080.7–2,120.0
Steps, n/d	21,409.5 (3,762.9)	19,213.6 (4,043.4)	.002	14,394.4–31,308.9	9,806.6–28,508.8

Abbreviations: MVPA, moderate to vigorous physical activity; NA, not applicable; SEDA, sedentary activity; SD, standard deviation.

a Differences between boys and girls were examined using *t* tests.

**Figure 2:**
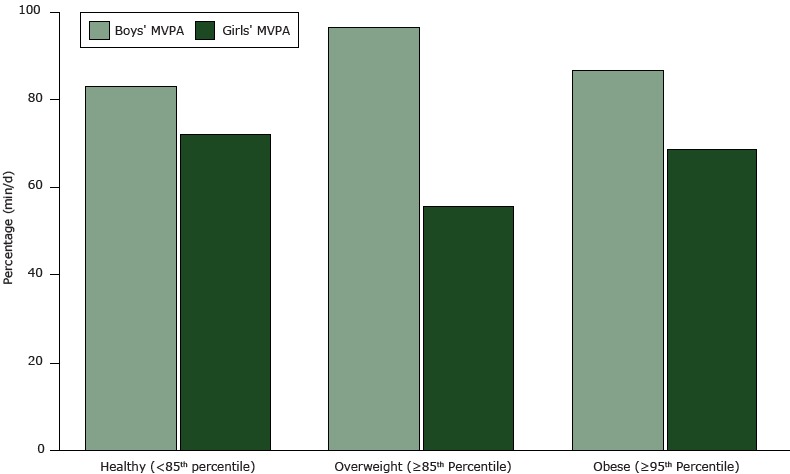
Average moderate to vigorous activity (MVPA) (min/d) by BMI status for boys and girls. Differences were significant among girls (*P* = .05). **Table:** 

Overall, children had an estimated average daily MVPA of 78 minutes. For all children, there was no significant association between BMI and MVPA. However, for girls, the average MVPA time per day was higher for those with a healthy BMI (72 min) than for those classified as overweight or obese (54 and 68 min, respectively; *P* =.05) (Figure 2). No difference was seen for boys.

Weight Status	Boys’ MVPA, min/d	Girls’ MVPA, min/d
Healthy (<85th percentile)	83.1	72.1
Overweight (≥85th to <95th percentile)	96.3	55.4
Obese (≥95th percentile)	86.5	68.6

Participants were classified into 2 BMI groups: healthy or overweight/obese status. Two children were classified as underweight and were grouped with children of healthy weight. Children with a healthy BMI had fewer average minutes of sedentary activity than did overweight/obese children (866.3 and 867.9 min/d, respectively; *P* < .05). A multivariate model was estimated with weight status as the dependent variable: independent variables were age, sex, MVPA, energy expenditure (kcal), steps, and sleep duration (Table 3). Higher MVPA was associated with an increase of 0.030 (adjusted odds ratio) in the likelihood of being in the healthy weight category. The adjusted odds ratio for overweight/obesity did not significantly vary by sleep or number of steps.

**Table 3:** Logistic Regression Model for Impact of Activity Levels on Weight Status, Niños Sanos, Familia Sana (Healthy Children, Healthy Family), Fresno Valley, California, 2012–2013

Variable	β	SE	Wald χ^2^	Adjusted Odds Ratio (95% CI)	*P* Value
**Healthy weight^a^**					
Age	0.795	0.267	7.593	2.08 (1.24–3.49)	.006
MVPA	0.032	0.013	5.646	1.03 (1.01–1.05)	.02
Steps	0	0	0.008	1.00 (1.00–1.00)	.93
Energy expenditure	−0.010	0.002	19.454	0.99 (0.99–0.99)	<.001
Sleep duration	0.005	0.004	1.491	0.99 (0.99–1.00)	.22
Sex	−1.060	0.486	4.004	0.38 (0.15–0.98)	.05
Constant	−11.497	3.894	8.590	—	—

Abbreviations: —, not applicable; β, beta coefficient; CI, confidence interval; MVPA, moderate to vigorous physical activity; SE, standard error.

a The reference category is overweight/obese and was defined using the Centers for Disease Control and Prevention’s growth charts as body mass index ≥85th percentile. Healthy weight was defined as BMI <85th percentile.

## Discussion

Our results, based on analyses of data collected from the Polar Active accelerometer and anthropometric measurements, suggest that the relationship between physical activity and weight status differs by both sex and age. Our regression analyses suggest that daily MVPA time has a significant negative association with a child’s likelihood of being overweight or obese. Conversely, other metrics of physical activity (ie, steps) did not demonstrate such a relationship. These results thus demonstrate the value in measuring both the quantity and quality of physical activity in the context of childhood obesity prevention. These results are similar to those of other studies that show MVPA to be correlated with child health indicators (ie, BMI, biochemical metabolites) (27–29) and deem lack of physical activity an independent risk factor for metabolic syndrome in children.

The baseline data reported in this article provide important information on the weight status and physical activity patterns of the program’s study population. The high prevalence of overweight and obesity (42.3% of children) is especially concerning considering the young age of the sample. At 23%, childhood obesity is higher in this study population than in the US general population; the US rate is 18% for children aged 6 to 11. This finding agrees with other evidence suggesting that Mexican American populations are at increased risk of obesity and chronic illness. Given these patterns, this study models the use of accelerometers to identify causal factors that may help explain this population’s relatively higher rate of overweight and obesity.

Accelerometry is a useful tool for the fields of health promotion and research because it can measure objectively the energy used by a person’s physical activity. The units are measured as metabolic equivalents of task, or METs. However, methodological differences between devices limit the relevance of accelerometer data outside the context in which they are collected (16–19). Varying cut points and thresholds across manufacturers and models result in differing estimates of physical activity intensity from different devices (16). The Polar Active, used in this study, has an inherently programmed threshold of ≥3.5 METs for determining MVPA time (25). Some researchers suggest adjusting for the higher resting energy expenditure of children by using thresholds of more than 4 or 4.5 METs (26). Thus, the lower threshold of the Polar Active technology probably results in a higher estimate of MVPA time than some other methods. The measured MVPA time for this study population appears higher than the national average (30). Instead of comparing these findings to guidelines or results from other studies, these data serve to examine associations between physical activity and other important health variables of children in this study population and to measure the impact of the intervention on children’s physical activity over time.

Additional limitations to using the Polar Active include the feedback features (Figure 1) on the device face, which may skew the user’s activity, although no reactivity was observed in this study. Our research team observed that child users were often excited about the animated feature on the accelerometer watch face and this feature may have aided in device compliance. Compliance is a particularly important feasibility issue related to the use of wearable electronic devices in large-scale field studies, especially studies of children. This study showed a 72% compliance rate, which is comparable to or higher than other studies conducted in child populations. This rate may also be attributed in part to the CBPR approach used as part of the NSFS project’s methodology, which allowed for the distribution and collection of devices from children in a manner that families found convenient. A contribution of this study is the use of accelerometer technology to generate useful health and physical activity data within this population, considered “hard-to-reach” in terms of socioeconomic, geographic, and linguistic barriers.

We selected the Polar Active for physical activity measurement in this study because of its proven feasibility for use with school-aged children. Further, the Polar Active proved more cost-effective than many other accelerometer devices, which (complete with accessories and analyses packages), ranged in price up to 10 times as much as the Polar Active (31). From a researcher’s perspective, the Polar Active system interface was time-consuming to set up for collecting and retrieving the data in a useable format. But because of the rapidly growing field of wearable technology, cost-effective wireless devices are becoming more commercially abundant, and many of these allow for easier set up and access to data.

In California’s Central Valley, childhood obesity is a significant threat to the health of the populations that live there. Effective promotion of MVPA recommendations is expected to help prevent childhood obesity. Moreover, identifying effective and easy methods to assess physical activity levels and patterns is important for assessing impact of future physical activity interventions among children within this target age and ethnic group. Given the paucity of empirical data that inform physical activity intervention effectiveness, accelerometers may be useful in assessing school-based and community-based physical activity intervention strategies, particularly for hard-to-reach populations such as this study’s population.
